# Pollution Assessment and SSD-Based Ecological Assessment of Heavy Metals in Multimedia in the Coast of Southeast China

**DOI:** 10.3390/ijerph192316022

**Published:** 2022-11-30

**Authors:** Rong Lu, Shaowei Rong, Jin Wu, Weifeng Yue, Qun Li

**Affiliations:** 1 College of Water Sciences, Beijing Normal University, No. 19 Xinjiekouwai Street, Haidian District, Beijing 100875, China; 2 South China Sea Marine Survey Center, Ministry of Natural Resources, Guangzhou 510275, China; 3 College of Architecture and Civil Engineering, Beijing University of Technology, Beijing 100124, China; 4 Ministry of Ecology and Environment Peoples Republic of China, Nanjing Institute of Environmental Science, No.8, Jiangwang Miao Street, Nanjing 210042, China

**Keywords:** environmental toxicology, heavy metal pollution, marine pollution, species sensitivity distribution, ecological risk assessment

## Abstract

Heavy metals in the ocean exist in various media and assessing heavy metal pollution in the multimedia in seawater is important for proposing effective strategies to protect marine ecosystem health. However, comprehensive coastal pollution assessments and SSD-based assessments of heavy metals have been limited from an international perspective. This study discusses the distribution, sources, interactions, associated environmental factors, and potential ecological risks related to heavy metal pollution. To attain this objective, several tools and models were considered. The partition coefficient between sediment and water was used to understand the ability of heavy metals to be adsorbed from seawater by sediment. The water quality index was applied to evaluate the comprehensive heavy metal pollution at different sampling sites. The species diversity index was calculated by calculating the concentration of chlorophyll a. The geo-accumulation and potential ecological risk indexes were employed for the sediments’ general pollution characteristic of heavy metals. Species sensitivity distribution was used for ecological risk evaluation. The results indicated that heavy metal pollution and ecological risk (Cu, Pb, Zn, Cd, As, Cr and Hg) are not serious, and the pollution conditions remain safe. Only Cu, Pb, and Hg concentrations in seawater exceed the Nation Class I Water Quality Standard. The concentrations of heavy metals showed significant spatial characteristics. Fisheries activities and industrial effluent discharges were identified as the main anthropogenic sources. This study provides a comprehensive assessment of heavy metals in multi-media, and the results will provide valuable information for nearshore ecological management and risk avoidance.

## 1. Introduction

As a dangerous pollutant in the ecological environment, heavy metal is famous for their high toxicity, non-biodegradability, and ease of accumulating rapidly in the environment [1]. The marine ecosystem is the largest and most directly tied to people’s lives. Heavy metals in ecosystems come from various sources, some of which are natural phenomena such as weathering and volcanic eruptions [2], most of which are human activities [3,4,5]. Heavy metals from industrial, agricultural, and domestic wastewater reach marine ecosystems via rainfall runoff and rivers, accumulate in the sediment and threaten the survival of aquatic organisms and the operation of marine ecosystems. Heavy metals that accumulate in sediments are difficult to remove and prevent from being released into seawater [6].

Another serious threat to marine ecosystems posed by heavy metal pollution is that exposure to heavy metals through seafood ingestion is often very obvious [7]. Ingested heavy metals can accumulate in vital organs such as the kidneys, bones, and liver, posing a threat to human health [8,9]. Marine heavy-metal pollution poses a significant health risk to humans due to the biomagnification effect, and the heavy metal pollution of marine products has gradually become a hot issue [10,11,12,13]. Therefore, it is of practical significance to study the content, distribution, and risk assessment of heavy metals in marine ecosystems to prevent marine ecological degradation and evaluate the pollution status of pollution in water.

Shantou, located in northeastern Guangdong, has rich fishery and tourism resources and plays a critical role in the economic development of coastal Guangdong [14]. Shantou City has several estuaries. The water environment of the estuaries at the intersection of the rivers and the ocean is complex, the sediment movement is intense, and the water environment is greatly affected by humans, which has typical dynamic characteristics. China’s famous Shantou Bay is located here. In recent decades, with Shantou’s economic development and urbanization, many inland pollutants have migrated to the estuary area along with the river and deposit, which has caused tremendous pressure on the aquatic ecological environment [15]. As Shantou is a special economic zone, the mining, the development of high-tech industry and the construction of reclamation projects have not only brought heavy metal pollution to the water environment system of Shantou’s sea area but also greatly compressed the area of the bay wetlands, reduced the self-cleaning ability of the environment [16], and caused significant pressure on the marine environment of the surrounding sea area of Shantou.

At present, many scholars in China only study the sediment or bioconcentration in Shantou Bay and the inner bay of Shantou [14,17,18,19,20], while the heavy metal pollution of seawater, sediments, and organisms in Shantou’s sea area is rarely studied. Therefore, after sampling and investigating the seawater, sediments and organisms in Shantou’s sea area, this study analyzed the distribution characteristics, pollution levels and possible sources of heavy metals by using various methods and models and evaluated the potential ecological risks of heavy metals. This study is of great significance to the prevention and control of heavy metal pollution in Shantou’s sea area and provides useful suggestions for the risk assessment of heavy metal pollution in Shantou and the sustainable development of marine ecosystems.

## 2. Materials and Methods

### 2.1. Study Area

**Figure 1:**
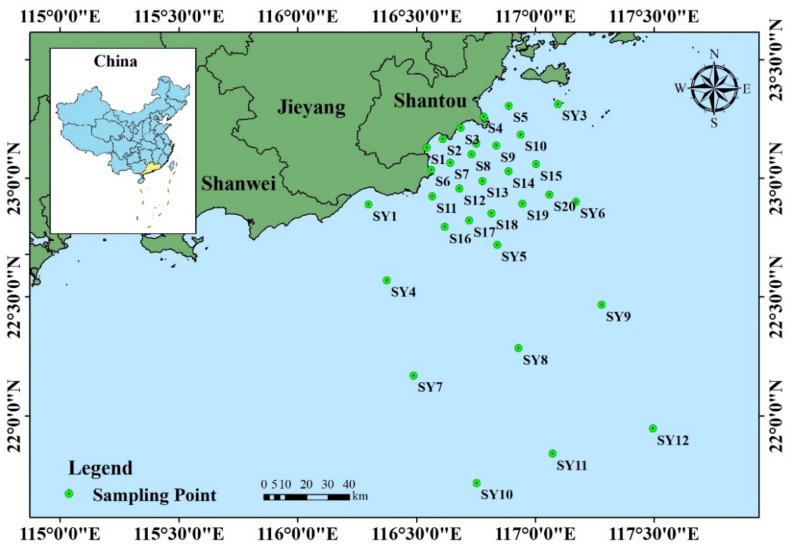
Study area and sampling sites.

Shantou City is located in the east of Guangdong Province, south of the Han River Delta (between 116°14′ and 117°19′ E and 23°02′ and 23°38′ N) (Figure 1). South of Shantou is the vast South China Sea, which is close to Hong Kong and Taiwan, i.e., its geographical location is very important. Shantou is an important transportation hub in East Guangdong, South Jiangxi and southwest Fujian, a port of entry and exit, a commodity distribution center, and an important gateway to the Maritime Silk Road. Shantou has seven districts and counties, with a total of 2199 square kilometers. The population density is 2518 people/km^2^, 4.5 times that of the whole province. Shantou’s landform is dominated by the delta alluvial plain, the largest plain in eastern Guangdong. The coastline of the Shantou mainland is 217.7 km long, and the coastline of the islands is 167.37 km long. The working area of the marine functional area of Shantou is about 10,000 km^2^, which is five times that of the land area. There are 82 islands of various sizes in Shantou.

Most of the territory of Shantou is subtropical, between the inter-tropical convergence zone and the subtropical high-pressure belt, in the south of the northeast trade wind belt. Shantou is located at the Eurasian continent’s southeast end, on the Pacific Ocean’s west coast, near the South China Sea. It often blows north in winter and south or southeast in summer, with obvious monsoon climate characteristics. The sea current in Shantou moves northeastward along the mainland in the spring, showing current barotropic characteristics. In the autumn, Shantou is in the transition season from the southwest monsoon to the northeast monsoon season, and the sea area near Shantou has a southwest current from surface to bottom. The Tropic of Cancer passes through the northern area of Shantou city. The annual rainfall ranges from 1300 to 1800 mm, mostly concentrated from April to September.

### 2.2. Sample Collection and Analysis

Twenty large-scale survey stations (S1–S20) were laid on the coast near Shantou, with a distance of about 5~10 km. A seawater quality survey was conducted at all stations. Sediment surveys were conducted at 12 stations. Marine organisms (including chlorophyll-a content, primary productivity, phytoplankton, plankton and benthic organisms) were investigated at 14 stations, and marine biological quality surveys were conducted at 12 stations (the stations were consistent with fishery resource surveys). At the same time, 12 stations (SY1–SY12) were set up to survey fishery resources and biological quality in Shantou waters (Figure 1).

The survey was carried out in April 2020 and September 2020 in the two seasons noted above. The survey was carried out in April with the fishing boat “Yuezhu Fishing 30081” and in September with the fishing boat “Yuezhu Fishing 30006”. The water quality survey was carried out by taking a water sample 1 m below the surface, taking a sample and filtering it through a 0.45 μm, Ø60 mm microporous filter membrane, and placing it in a clean polyethylene bag in a cooler at 4 °C. Another water sample was filtered through a 0.45–0.7 μm, Ø47 mm glass fiber filter, placed in a clean polyethylene bag in a cooler at 4 °C and taken back to the laboratory for analysis of chlorophyll concentrations. Sediment samples were collected from 0–10 cm below the surface of the seafloor sediment using a Van Veen grab. After sampling, the sediment samples were sealed in clean polyethylene bags, placed in a cooler at 4 °C, and transported to the laboratory for further analysis, along with the water samples taken.

Phytoplankton samples were sampled with shallow water type III plankton net or small plankton net, and zooplankton samples were sampled with shallow water type I plankton net or large plankton net, and the samples were fixed and stored in 5% formalin seawater solution. The samples were stored in a 5% formalin seawater solution. Individual counting methods identified the samples. The benthic samples were collected by using a 0.05 m^2^ eosinophilic mud collector (more than two buckets per station) to obtain mud samples rinsed with 1 mm sieve, and then all biological individuals were picked out and fixed with 5% formaldehyde solution.

Biological samples were collected by trawl or grab sampler at stations SY1–SY12. Approximately 100 g of muscle tissue of fish and crustaceans and edible parts of mollusks were taken, washed, and placed in clean polyethylene bags, sent to the laboratory, and then the samples were freeze-dried for 24 h and crushed.

The heavy metal contents of Hg and As were analyzed by atomic fluorescence spectrophotometry (AFS), Cu, Pb, Cd and Cr by flameless atomic absorption spectrophotometry, and Zn by flame atomic absorption spectrophotometry [21,22]. The seawater samples used to measure chlorophyll-a concentration were extracted with a 90% acetone solution of phytoplankton pigments, and the absorbance values were measured at 664 nm, 647 nm, 630 nm and 750 nm in that order. All analytical data are subject to strict quality control. Data processing and quality control of analysis was carried out according to the certified reference material of the State Oceanic Administration (GB 17378.2-2007). The recovery rates of heavy metals varied between 86% and 95%, and the differences in metal concentration between the research results and the certified values were generally less than 10%.

### 2.3. Assessment Methods

#### 2.3.1. The Transport and Fate of Heavy Metals in Seawater and Sediments

To study the distribution of heavy metals in the water ecology, it is necessary to study the ability of heavy metals to be adsorbed from the liquid phase (seawater) by the solid phase (sediment). This capacity is expressed as a partition coefficient (*K_d_*) between sediment and water. This method is widely used in the field or laboratory to determine the transport capacity of heavy metals [23,24,25]. The *K_d_* is calculated as follows.

(1) $$K_{d}=\frac{C_{s}}{C_{d}}$$

where *C_s_* is the concentration of heavy metals in the solid state (sediment), mg/kg. *C_d_* is the concentration of heavy metals in the liquid phase (seawater), mg/kg.

#### 2.3.2. Marine Pollution Assessment

There are many methods to study heavy metal pollution in aquatic ecosystems, such as the exponential method system and the fuzzy mathematical method (also called the gray method). In this paper, the method of pollution factor (*C_f_*) proposed by Hakanson in 1980 was used to evaluate the single indexes [26]. The comprehensive water quality evaluation was evaluated using the integrated index *WQI* method [27]. The formula is as follows.

(2) $$C_{f}=\frac{C_{s}}{C_{b}}$$

(3) $$WQI=\frac{1}{n}\sum_{i=1}^{n}C_{f}$$

The *C_f_* index indicates the pollution degree of a single metal; *C_s_* and *C_b_* are the target metal concentration at the location and the standard concentration of the same metal in the selected reference background, respectively. *WQI* is the water quality index, *n* is the number of items of all the heavy metals involved in the evaluation, *WQI* < 1 means low pollution; 1 < *WQI* ≤ 2, medium pollution; 2 < *WQI* ≤ 3, serious pollution; *WQI* > 3, very serious pollution.

#### 2.3.3. Biological Quality Assessment

This study calculated the chlorophyll-a content according to the Jeffrey–Humphrey’s equation and expressed in mg/m^3^ [28]. The CADEE (1975) equation was used to estimate primary productivity based on chlorophyll a, transparency, water depth, light time and carbon assimilation coefficient [29]. Biological quality was evaluated by the primary productivity and biodiversity index.

(4) $$\mathrm{Chla}=11.85\times {(E}_{664}{-E}_{750})-1.54\times {(E}_{647}{-E}_{750})-0.08\times {(E}_{630}{-E}_{750})$$

(5) $$P=\frac{C_{a}QLt}{2}$$

(6) $$H^{\prime }=-\sum_{i=1}^{S}P_{i}\log_{2}P_{i}$$

Chla is the chlorophyll-a concentration, mg/m^3^, and E_750_, E_664_, E_647_ and E_630_ are the absorbance values at 750, 664, 647 and 630 nm. *p* is the primary productivity, mg·C/(m^2^·d); *C_a_* is the chlorophyll-a concentration, mg/m^3^; *Q* is the assimilation coefficient, mg·C/(mgChl-a·h), here taken as 3.7 [30]; *L* is the depth of the euphotic zone; *t* is the day length in hours from sunrise to sunset. *H*′ is the species diversity index, *S* is the number of species in the sample, and *P_i_* is the number of the *i*th species to the total number of species ratio.

#### 2.3.4. Species Sensitivity Distribution Assessment

Ecological risk assessment is developed from risk assessment, and after decades of development, its evaluation content, scope, and scale have changed greatly. Compared with the previous study scale, which was single and the study object was only terrestrial, the study scale of ecological risk assessment nowadays is extended to ecosystems, and the study object is also extended from terrestrial to marine [31,32,33]. The species sensitivity distribution method is widely used because of its simplicity and clear ecological significance [34,35]. Currently, there are no theoretical studies to prove that SSD belongs to a particular curve form, so different fitting methods can be chosen [36]. The normal distribution, logistic distribution, lognormal distribution, and log-logistic distribution are recommended for China [37]. The logistic distribution is a two-parameter continuous-type probability distribution, similar to the normal distribution. Compared with the normal distribution, the logistic distribution is prone to extreme values, and the generalized logistic distribution is obtained by adding two shape parameters to the original function, which can describe the tail of the curve in detail [38,39].

(7) $$F(x)=\frac{e^{\frac{x-\mu }{\sigma }}}{\sigma {\left(1+e^{\frac{x-\mu }{\sigma }}\right)}^{2}}$$

(8) ln(HC5)=μ−1.6234σ

(9) $$msPAF=1-\Pi_{i=1}^{n}\left(1-PAF_{i}\right)$$

*x* is the toxicity value, mg/L; *μ* is the mean value of the toxicity value, mg/L; and *σ* is the shape parameter, mg/L. The pollutant concentration corresponding to a 5% cumulative probability on the SSD fitted curve is *HC5*; *PAF* indicates the proportion of species whose environmental concentration exceeds the biotoxicological endpoint value, i.e., the cumulative probability corresponding to a given measured pollutant concentration on the SSD curve; the values of *HC5* and *PAF* are directly readable on the SSD curve. Both *HC5* and *PAF* values can be read directly on the SSD curve. *msPAF* reflects the combined contamination of multiple pollutants in the water body.

#### 2.3.5. Geochemical Methods

This study evaluated sediment heavy metal concentration levels using the geo-accumulation index (*I_geo_*) and potential ecological risk index (PERI). In his 1969 study, Muller proposed *I_geo_* as a geochemical evaluation of metal contamination in sediments criteria [40], defined as:

(10) $$I_{geo}=\log_{2}(C_{n}/1.5C_{b})$$

*C_n_* is the measured concentration of trace metals in the sediment (mg/kg); *C_b_* is the geochemical background concentration of the corresponding metal (mg/kg); and a factor of 1.5 is used to respond to very small anthropogenic influences. According to Muller (1981), pollution conditions can be classified into seven classes: *I_geo_* < 0, uncontaminated; 0 < *I_geo_* ≤ 1, lightly contaminated; 1 < *I_geo_* ≤ 2, moderately contaminated; 2 < *I_geo_* ≤ 3, moderately to heavily contaminated; 3 < *I_geo_* ≤ 4, heavily contaminated; 4 < *I_geo_* ≤ 5, heavily to extremely contaminated; and *I_geo_* >5, extremely contaminated [41].

Hakanson established the PERI method in 1980 to broadly assess the intensity of metal contamination in sediments by considering environmentally relevant metal toxicity and its response to ecological function [26], calculated as:

(11) $$PERI=\sum_{i=1}^{n}E_{r}^{i}=\sum_{i=1}^{n}T_{r}^{i}\times C_{f}^{i}$$

(12) $$E_{r}^{i}=T_{r}^{i}\times C_{f}^{i}$$

(13) $$C_{f}^{i}=C^{i}/C_{n}^{i}$$

$E_{r}^{i}$ is the individual indicator for element i, and $T_{r}^{i}$ is the toxicity response factor for element *i*. The $T_{r}^{i}$ values for the elements studied were Cu = 5, Pb = 5, Zn = 1, Cd = 30, Hg = 40, As = 10 and Cr = 2 [42]. *C^i^* is the measured content of heavy metal *i*, and $C_{n}^{i}$ is the background value of heavy metal *i* content. The background values for each element are shown in Table S1 [42]. $E_{r}^{i}$ can be classified into five categories, and PERI can be classified into four categories, as shown in Table S2 [43,44].

## 3. Results and Discussion

### 3.1. Characteristics of Heavy Metals in Seawater, Sediment and Marine Organism

#### 3.1.1. Contamination Characteristics of Heavy Metals in the Seawater

The results of the calculation of seawater, sediment, and organisms by the pollution factor (*C_f_*) method are shown in Table 1. From Table 1, it can be seen that the concentrations of Zn, Cd, As, and Cr in the spring and autumn seawater and the concentration of Cu in the autumn seawater did not exceed the concentrations required by the first class of the People’s Republic of China Seawater Quality Standards (GB3097-1997) (Table S3). The exceedance rate of Cu, Pb and Hg in the spring seawater was 5%, and only one site of all seawater sampling sites in the spring exceeded the target heavy metal content. In the fall, there were seven sites with Pb concentrations exceeding the first class of seawater quality standards, with an exceedance rate of 35%; there were three sites with Pb concentrations exceeding the first class of seawater quality standards, with an exceedance rate of 15%. However, the water quality of the sites with excessive heavy metal content meets the second class of seawater quality standards, which indicates that although there is a certain amount of heavy metal pollution near the coast of Shantou, this pollution is in an acceptable range.

**Figure 2:**
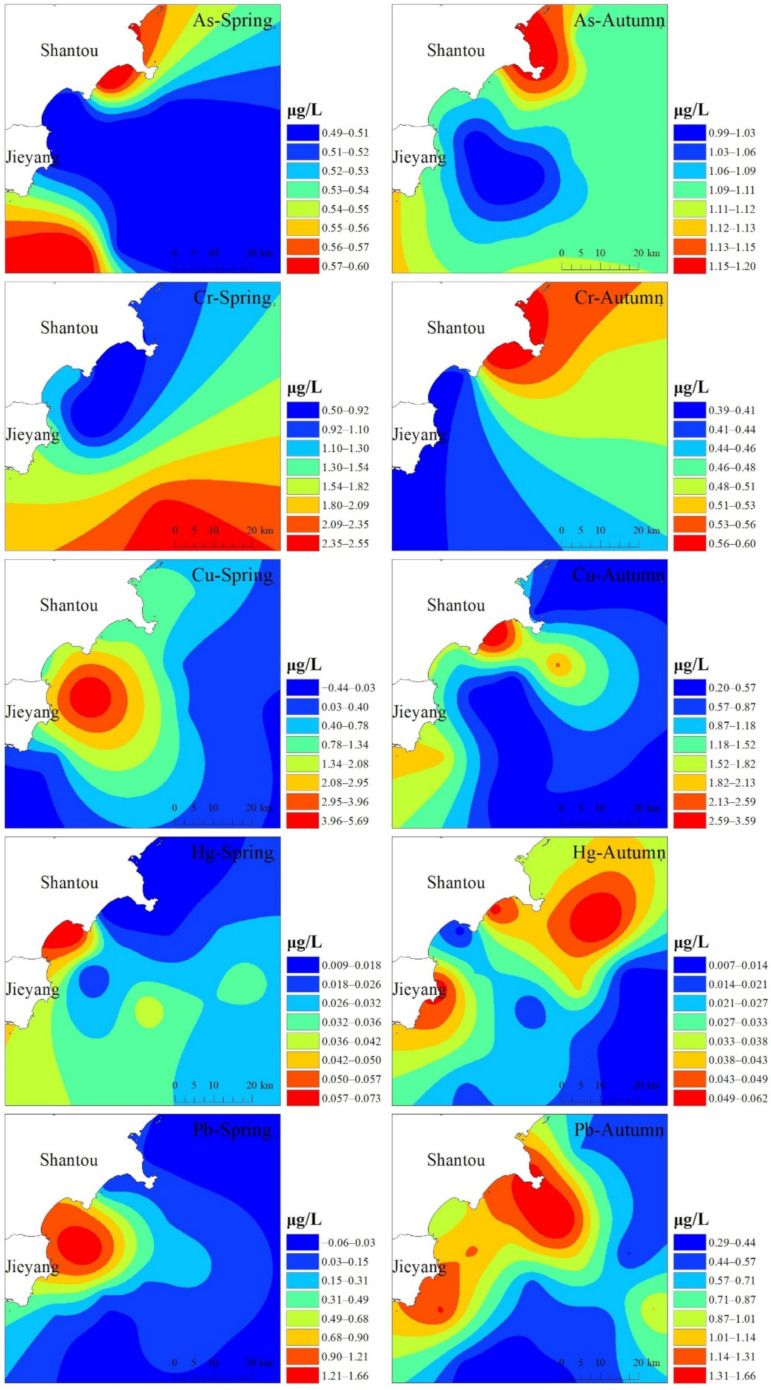

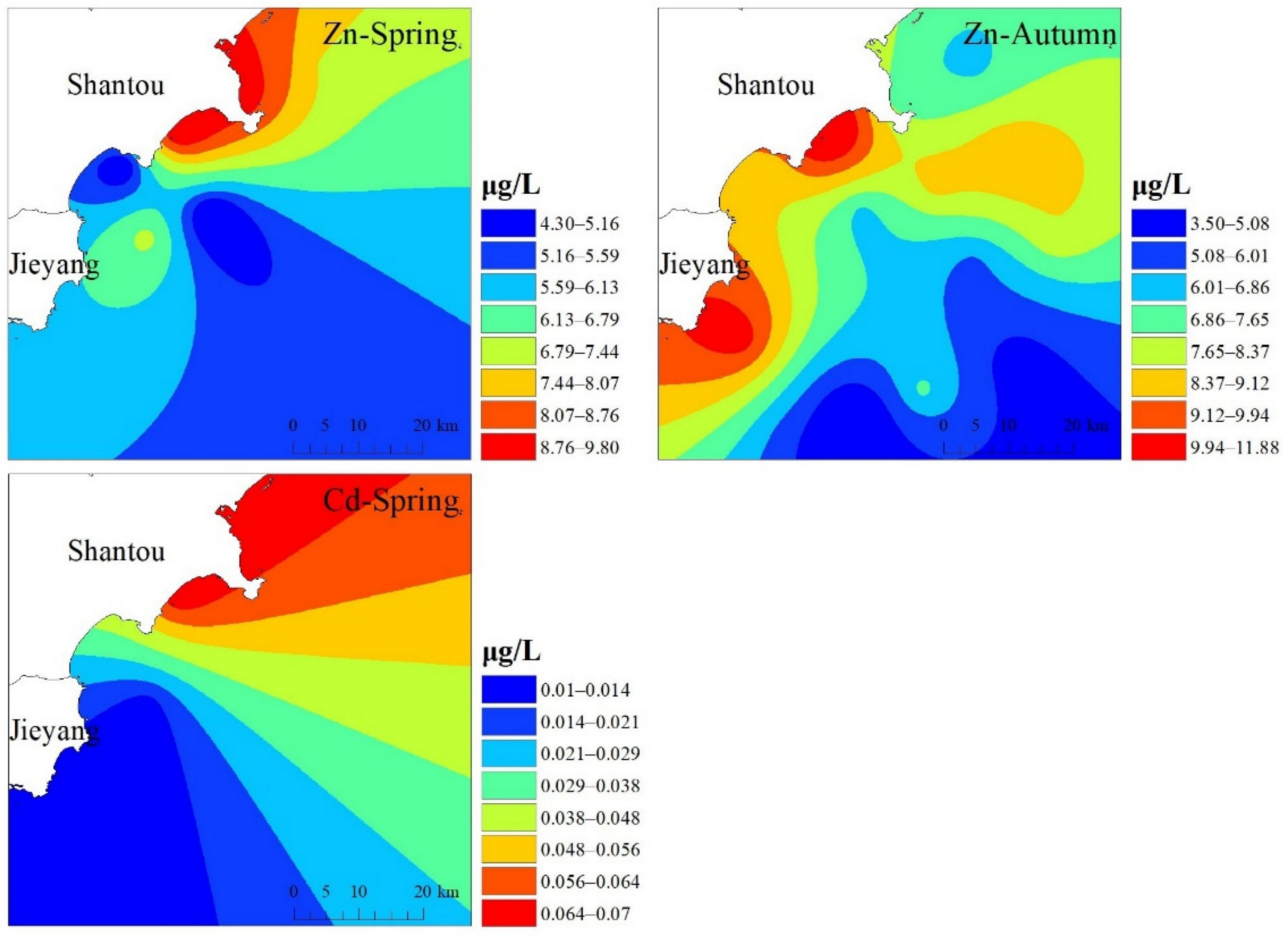
The spatial distribution of heavy metals in seawater.

The spatial distribution characteristics of heavy metals in the near coast of Shantou were obtained by interpolating the heavy metal contents in the study area with the Kriging method of Arcgis, as shown in Figure 2. Since Cd was not detected in any of the autumn seawater samples, which indicated that the Cd content in Shantou seawater was very low and did not exceed the standard, the situation of Cd in the autumn seawater was not analyzed, while in the spring seawater, since the concentration data of Cr and Cd in the spring seawater samples were not detected to be higher, the interpolation results may not be particularly accurate. Cr and Cd concentration data were not detected to be higher, so the interpolation results may not be entirely accurate. In the temporal dimension, the concentrations of all heavy metals, except Cu and Cr, were greater in the autumn than in the spring, which is likely due to the monsoonal climate of the coastal area of Guangdong [45]. The concentrations of Cr and Hg showed greater differences between the spring and autumn at the same points. 

Spatially, the distribution of heavy metals along the coast of Shantou showed a clear wave-like pattern. The distribution characteristics of As, Zn and Cd were most similar in the spring, with the lowest values occurring in the northern part of the Shantou coast and gradually increasing from north to south, while the distribution characteristics of Cr and Hg showed a gradual decrease from north to south. In the autumn, the overall distribution trend of heavy metal concentrations increased from the coast to the far side of the ocean, with Cu, Pb and Zn showing the most obvious presence. Among the seven heavy metals, the distribution of Cr and Hg is basically the same in the spring and autumn, but the concentration of each point varies greatly. The results of the overall water quality evaluation in Shantou’s sea area are shown in Figure S1. It can be seen from the figure that, except for S2 and S7, the indexes of all the stations in the autumn are larger than those in the spring, indicating the pollution levels of heavy metals in Shantou’s sea area are obviously lower in the spring. The spring sampling time is just after the Chinese Spring Festival. The activities of various enterprises are not active and may produce less pollution. August is generally the end of the fishing moratorium for fishermen, and the autumn sampling time is September, which is the peak of fishermen’s fishing activities. Most fishermen have an arbitrary discharge of domestic sewage. Ballast water or sewage can lead to higher heavy metal content, resulting in differences in the marine environment between autumn and spring. On the whole, the heavy metal levels in the nearshore stations (S1–S11) were higher than those in the far-shore stations (S12–S20). The water quality index of Shantou seawater is less than 1, which indicates that the pollution level of heavy metals is low at all stations in Shantou seawater, and although the pollution level is not high, it is still necessary to prevent the spread of heavy metal pollution.

#### 3.1.2. Contamination Characteristics of Heavy Metals in Sediments

The quality evaluation results of the marine sediments near Shantou calculated by the pollution factor method are shown in Table 1. The exceedance rate of heavy metals in the sediments of the investigated sea area was 0 at each site. Among them, the single mass indexes of Pb and Hg were larger at site S4, located at the outlet of Rongjiang River in Shantou at the junction of salt and fresh water. Therein, the heavy metals come from a wide range of sources, not only from surface runoff and urban sewage but also from atmospheric deposition and offshore input, which lead to the elevation of heavy metal content, and the developed water system provides convenient conditions for the transport of pollutants [18]. The tidal action is relatively strong in the eastern sea area of Shantou, and seawater can surge inland along the Rongjiang River, which is a harbor tidal channel with complex changes in channel dredging and hydrodynamic conditions. Suspended sand and biological debris carrying metals will stay and settle in the eastern sea area of the Haojiang River area under the action of the upwelling tide and sand barrage, forming a convergence at the end of the sand barrage [20]. This may be the reason for the high values of Pb and Hg in this area.

The results of the geoaccumulation index and potential ecological risk index calculations are shown in Table S4 and Figure S2. The calculation of *I_geo_* shows that the sea area near Shantou is not polluted by As, Cd and Cr (*I_geo_* < 0), and the pollution levels of the remaining heavy metals is not high; the average value of *I_geo_* of all heavy metals is less than 0, which is in the unpolluted state, the *I_geo_* value of Hg at point S4 is 2.63, which makes this point in moderate pollution. Overall, the pollution status of the Shantou coast has obvious spatial characteristics, and the *I_geo_* of Cu, Pb, Zn and Hg are all less than 0 at the point S13–S20, which indicates that the heavy metal pollution decreases gradually with the dispersion of the sea current. The average $E_{r}^{i}$ is in descending order: Hg > Cd > Pb > As > Cu > Cr > Zn, and it is worth noting that the risk of all heavy metals is low, except Hg, which is at considerable risk (*Ei r* > 80 and <160), and is mainly caused by the high content of Hg at points S3 to S11, especially S4. This is mainly due to the high content of Hg at points S3 to S11, especially at point S4, which makes a significant contribution to the evaluation of the pollution status of the sea near Shantou, which is related to the fact that point S4 is located at the outlet of Rongjiang River. When Cd was evaluated by *I_geo_*, it showed no contamination with Cd. However, the $E_{r}^{i}$ values for Cd were high, which may be due to its high toxicity response factor. The cumulative $E_{r}^{i}$ was used to calculate the PERI of the study area’s sediments, ranging from 27.35 to 431.98, with a mean value of 130.67. In terms of PERI, all sites in the study area were in low risk (<150) category. However, due to the unique geographical location of Shantou, future sewage discharge and fishing activities may increase the levels of anthropogenically derived metals, resulting in a PERI above the low-risk range.

#### 3.1.3. Contamination Characteristics of Heavy Metals in Marine Organisms

The standards for evaluating the quality of marine organisms along the coast of Shantou were adopted from the Concise Regulations for Comprehensive Investigation of National Coastal and Tidal Flat Resources and the People’s Republic of China Marine Biological Quality (GB 18421-2001). The results of the pollution factor method to calculate the pollution level of marine organisms are shown in Table 1.

In the spring survey, 12 samples of 6 species of fish, 3 species of crustaceans, 2 species of shellfish and 1 species of cephalopods were collected at 10 stations from SY1–SY9 and SY12. From the survey and monitoring results, the detection rate of Zn and Cd was 100%, Hg was 66.67%, Cu was 25.00%, Pb was 8.33% and As was 91.67%. The content of Hg and Pb was higher than that of other species.

The fall survey sampled 10 species of fish, 3 species of crustaceans, and 2 species of cephalopods at 12 stations from SY1–SY12. A total of 15 samples of 15 species were collected, and the results are shown in Table S5. Hg fish were higher than other categories; the Cu, Zn, Cd and As content in the crustaceans were higher than other categories.

Overall, among the collected marine organisms, the levels of Hg, Cu, Pb, Zn, Cd and As of fish, crustaceans and shellfish (mollusks) organisms all met the corresponding evaluation standards. The quality of organisms in the surveyed seas in the spring and autumn was good, and all factors relating to cephalopods did not exceed the standards.

### 3.2. Marine Organism Quality Assessment

**Figure 3:**
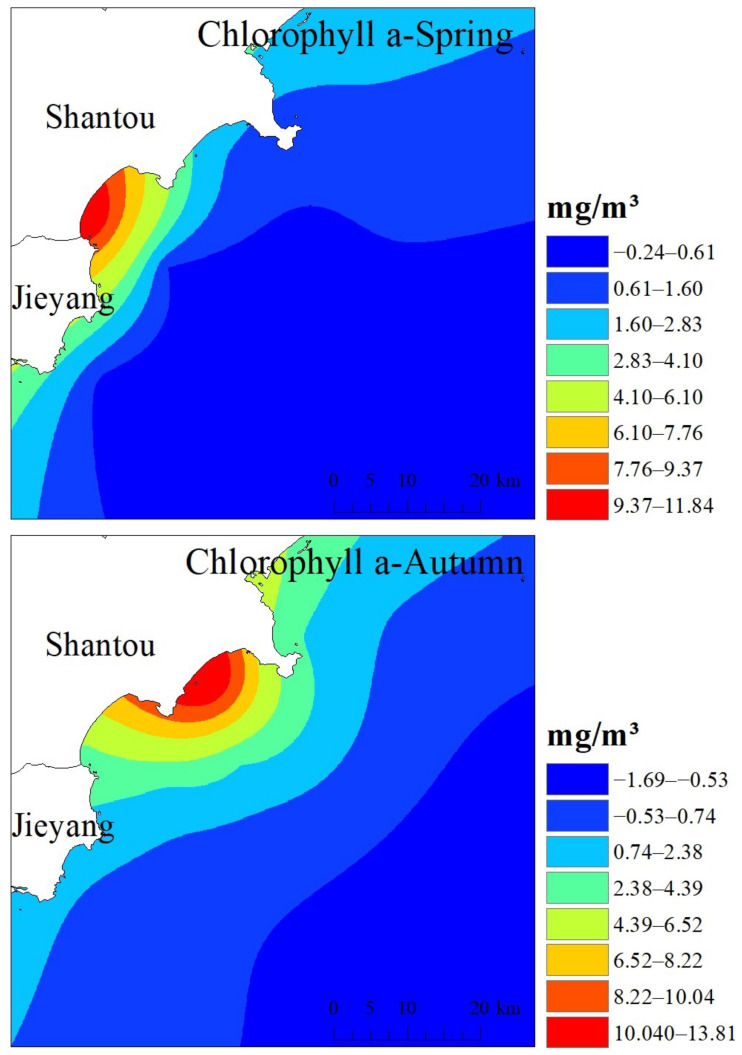
Results of chlorophyll a in coastal Shantou.

The marine ecological status survey of Shantou’s nearshore is shown in Figure 3. Referring to the relevant USEPA standards: ρ(Chla) > 10 mg/m^3^, the water body is eutrophic; 4 mg/m^3^ < ρ(Chla) < 10 mg/m^3^, mesotrophic; ρ(Chla) < 4 mg/m^3^, depleted. Chlorophyll-a samples were collected from 14 stations in the spring, and the chlorophyll-a mass concentration of each sample varied from 0.07 to 11.85 mg/m^3^, with a mean value of 1.51 mg/m^3^. Among the samples taken, the chlorophyll-a concentration of all stations was less than 4 mg/m^3^, except for the sample at S1, where the chlorophyll-a concentration was 11.85 mg/m^3^, and a mean value was also less than 4 mg/m^3^. The variation of marine primary productivity at each station near the coast in the spring ranged from 30.07 to 532.20 mg·C/(m^2^·d), with a mean value of 100.96 mg·C/(m^2^·d). In the autumn, sampling was conducted at the same stations as in the spring, and the chlorophyll-a mass concentration at each station varied from (undetected~13.85) mg/m^3^ with a mean value of 2.99 mg/m^3^. Except for the sample chlorophyll-a concentration of 13.85 mg/m^3^ at station S3, the chlorophyll-a concentration at all stations was less than 4 mg/m^3^, and the mean value of 2.99 mg/m^3^ was also less than 4 mg/m^3^. The variation of marine primary productivity in the nearshore section in the autumn ranged from 44.93 to 1166.72 mg·C/(m^2^·d), and the average value was 208.28 mg·C/(m^2^·d). Therefore, the chlorophyll a in the surveyed seas was at a poor nutrient level in both spring and autumn. As shown in Figure 3, the distribution trends of chlorophyll-a content in the spring and autumn were generally the same in Shantou’s near-coast area, with an obvious wave-like distribution and gradually decreasing from the near-coast to the far-coast. The highest values of S1 and S3 in the spring and autumn were located in Haimen Bay and Tangbian Bay, respectively. The phytoplankton living in the estuary and the bay area are provided with abundant basic nutrients for their growth and reproduction, so again, the chlorophyll-a concentrations are relatively high at sites S1 and S3. From the figure, there is an obvious northward trend in the autumn relative to spring, and a decreasing trend toward the southeast, which may be related to the tidal action and changes in hydrodynamic conditions.

**Figure 4:**
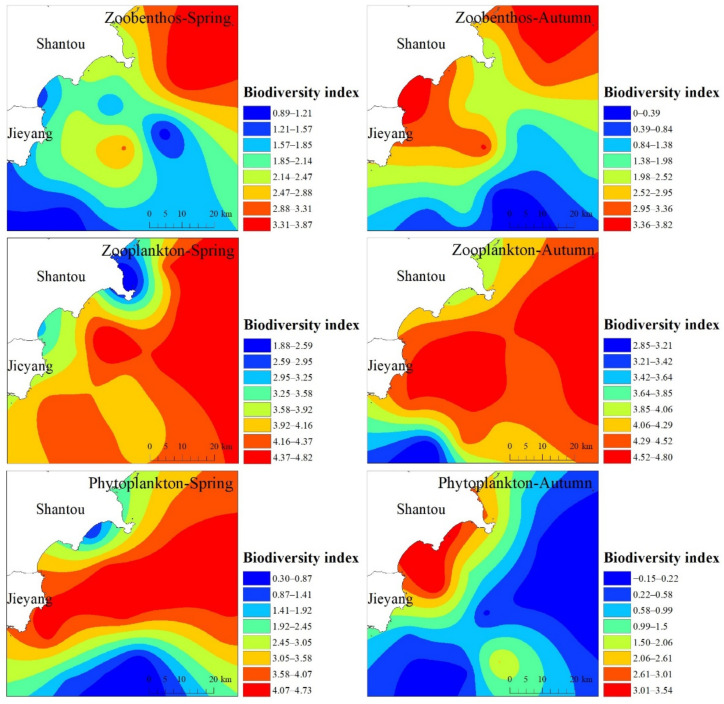
Evaluation results of species diversity index on the coast of Hong Kong.

The interpolated biodiversity index of Shantou nearshore is shown in Figure 4. 14 stations were surveyed for benthic organisms in the spring. A total of 53 species of macrobenthos were recorded, including 30 species of annelids, 7 species of mollusks, 6 species of arthropods, and 11 species of other animals (including 7 species of echinoderms, 1 species of neophyte, 1 species of cephalopods, and 2 species of chordates). The number of macrobenthic species at each quantitative sampling station in the survey area varied from 2 to 18 species/station, with an average of 6 species/station. The diversity index (*H*′) varied from 0.614 to 3.873, with a mean value of 2.021. The highest diversity index was recorded at station S10 and the lowest at station S20, with a high level of diversity. A total of 61 species of macrobenthos were recorded in the autumn survey, including 36 species of annelids, 4 species of mollusks, 12 species of arthropods, and 9 species of other animals (including 3 species of echinoderms, 1 species of stellate animals, 1 species of nematodes, 1 species of cephalopods, and 3 species of chordates). The number of macrobenthic species occurring at the sampling stations varied from 1 to 16 species/station, with an average of 7 species/station. The diversity index (*H*′) varied from 0.000 to 3.822, with a mean value of 2.277. The highest diversity index was found at station S1 and the lowest at station S18, with a medium level of diversity. The distribution of benthic diversity indices was similar in the spring and autumn, with an overall high in the north and low in the south and a high value in the autumn in the southern coastal area of Shantou that was not present in the spring, which may be related to the freshwater input and salinity changes caused by tidal movements in this area [46].

A total of 111 species of 13 zooplankton taxa were recorded in the spring zooplankton survey, and the most abundant species were copepods, with 47 species. The average number of zooplankton species in each station was 43 (5~59 species); the species diversity index ranged from 1.880 to 4.815, with an average of 4.017. The highest diversity index was found in sampling station S8, followed by S10, and the lowest was found in sampling station S4, with a high level of diversity. A total of 110 species of zooplankton from 14 taxa were recorded in the fall survey, and the most diverse taxa were copepods with 54 species, as in the spring. The average number of zooplankton species in each station was 48 (33–60 species); the species diversity index ranged from 2.855 to 4.800, with an average of 4.302. The highest diversity index was found in sampling station S13, followed by S10, and the lowest was found in sampling station S16, with a high level of diversity. From the average occurrence of zooplankton species and the biodiversity index of each station, the biodiversity in the autumn had a slight increase compared with that in the spring. The distribution of zooplankton diversity indices was roughly the same in the spring and autumn, with a gradual increase from the coast to the ocean depth.

A total of 60 genera and 176 species (including 3 varieties and 2 variants) in 4 phyla were recorded in the spring phytoplankton survey. The diatom phylum appeared as the most abundant species, with 43 genera and 112 species. The number of phytoplankton species varied from 41 to 85 species at each station, with an average of 63 species. The Shannon–Wiener diversity index ranged from 0.299 to 4.651, with an average of 3.050. The diversity index was highest at station S8, followed by station S13, and lowest at station S18, at 0.299. A total of 176 species in 60 genera of 5 phytoplankton phyla were recorded in the autumn survey (including 3 varieties). The fall survey recorded 176 species (including 3 varieties and 2 variants) in 5 phyla. Among them, diatoms were the most abundant species, with 39 genera and 101 species accounting for 57.39% of the total number of species. The number of phytoplankton species varied from 47 to 78 species at each station, with an average of 63 species. The Shannon–Wiener diversity index ranged from 0.054 to 3.536, with an average of 1.275. The diversity index was highest at station S7, followed by station S3, and lowest at station S16. The diversity index was at a low level. It can be seen from the figure that the distribution of phytoplankton is not the same in the spring and autumn, and the area with high values in the biodiversity index increases significantly in the spring when the temperature increases. The combined effect of surface runoff and tides in estuarine bays and other places along the coast of Shantou makes the water body fully mixed and rich in nutrient salts, which is conducive to the proliferation of phytoplankton such as algae, which is also an important reason for the high-value area of chlorophyll-a content in the sea near the estuary in the spring. This is also an important reason for the significant expansion of the area with high levels of chlorophyll a.

### 3.3. Interrelation and Source Analysis of Heavy Metals

**Figure 5:**
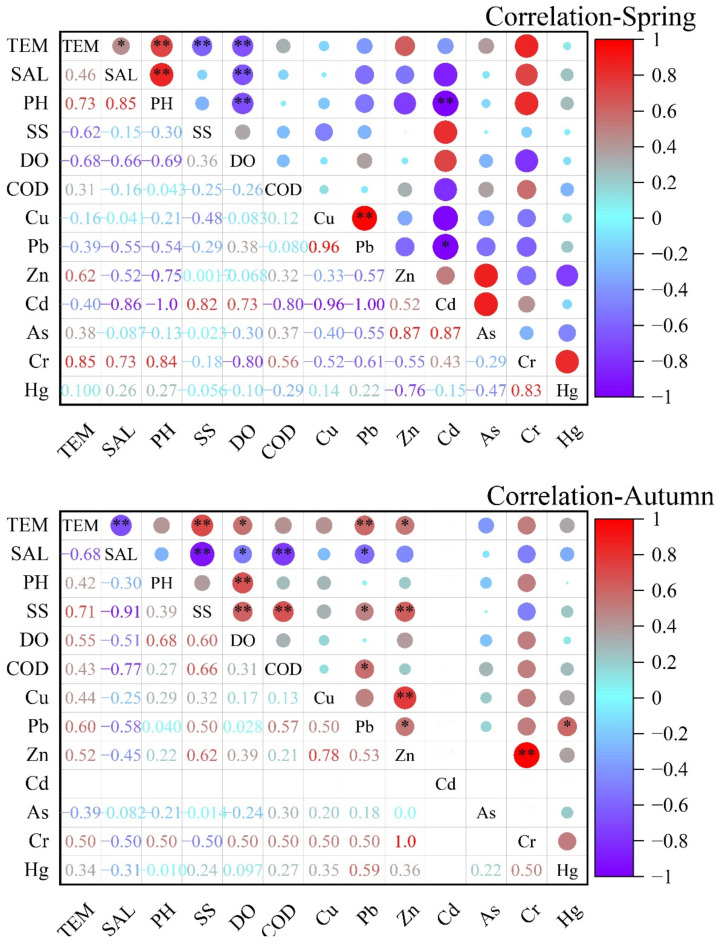
The correlation analysis of heavy metals in the seawater of coastal Shantou (* *p* < 0.05; ** *p* < 0.01).

The results of the correlation analysis between heavy metals and other particulate matter and heavy metals in Shantou’s sea area are shown in Figure 5. From the correlation matrix, we can see that in the spring, the correlation between Cu and Pb is the highest, and the correlation between Cu and Pb and other heavy metals except Hg is negative, up to 0.96, while in the autumn, the correlation between Cu and Pb decreases to 0.5, which indicates that Cu and Pb probably have the same. This indicates that Cu and Pb probably have the same source of pollution and are not the same as other heavy metals, but the correlation decreases due to the seasonal production activities, which leads to lower emissions of a certain heavy metal in the autumn. The correlations of Cd with Cu and Pb in the spring were −0.96 and −1, indicating that Cu and Pb had completely different sources from Cd. Cr had good correlations with temperature and PH in the spring and autumn, indicating that Cr’s dissolution in seawater was closely related to seawater temperature and PH. Overall, the seven heavy metals in the spring seawater do not have homology among themselves; Cu and Pb have homology, Zn, Cd, and As have a higher possibility of having the same source, and Cr and Hg also have some homology. In autumn seawater, all seven heavy metals have some correlation; that is, there is some homology among the seven heavy metals. Considering the discharge from the coastal rivers in the east of Shantou, the development of coastal industries, and the distribution of industrial structures in towns, the source of the heavy metals in the coastal area of Shantou is considered to be anthropogenic, which reflects that industrial wastewater and urban discharge are the main sources of heavy metals in marine sediments. The difference in correlation between heavy metals in the spring and autumn is due to the seasonal production activities that lead to different emissions of different heavy metals in different seasons.

*K_d_* is used to express the partition coefficient between sediment and water, which can visually reflect the migration ability of heavy metals from the liquid phase to the solid phase. Its main influencing factors are the characteristics of heavy metals and the changes in the liquid phase and solid phase. The results of partition coefficients (K_d_) in Shantou seawater are shown in Table 2. Since there were more undetected data in the spring seawater as well as the Cd undetected in the autumn, an analysis of the migration ability of Cd was not carried out at the time, and only the existing data were relied upon to analyze the migration ability of heavy metals. 

Comparing the available data, it was found that the K_d_ values of Pb in the spring were significantly higher than those in the autumn, which indicated that Pb was more likely to be transferred from seawater to accumulate in sediment in the spring, and this phenomenon also occurred in Hg and As at the same stations, which suggested that the migration ability of the heavy metals in sediment and seawater might be influenced by temperature. Except for Pb, the high values of K_d_ are generally found in the sea area nearer to the coast. This phenomenon indicates that heavy metals are more easily enriched by sediments near the coast, while in the deeper water area away from the coast, they are more difficult to be enriched by sediments due to the movement of ocean currents, but the partition coefficients are greater than one except for a very few numbers of points, which indicates that only a small portion of the free metal ions are dissolved in water. Although heavy metals are not degraded in water, they are not usually present at high concentrations because they are deposited by sediments and absorbed by marine plants and animals in seawater [47].

Shantou is one of the earliest special economic zones in China and has a variety of industries, such as textiles and garments, chemicals and plastics, craft toys, printing and packaging, and other traditional advantageous industries. Zhelin Bay in the Shantou area is an intensive mariculture area. Therefore, industrial and agricultural wastewater is an important source of metals in the area [13]. In addition, Hanjiang, as the second largest river in Guangdong Province, flows through the Chaoshan Plain and important industrial cities, such as Chaozhou and Jieyang, and into the South China Sea in Shantou City as the source, bringing a large number of pollutants to the coastal area [13]. In addition to the reasons noted above, the large loss of coastal wetlands in Guangdong is also an important reason for the accumulation of heavy metals in the ocean, especially in the bay wetlands, which have an extremely strong self-purification capacity. From 1985 to 2005, many areas in eastern Guangdong, including Shantou, added 468,000 km^2^ of reclamation, of which the fisheries accounted for 29.2% and construction for 23.4% of the total [11]. At the same time, since Shantou became a special economic zone, the large population influx has led to accelerated urbanization, the rapid development of agriculture and fishery industries, and increased land use for construction. In addition, reclamation projects have also weakened the seawater dynamics, reduced the area of wetlands in the bay, and decreased the self-purification capacity of seawater, which eventually caused heavy metals to remain in the seawater and be enriched by sediments.

### 3.4. Hazard/Risk Assessment

**Figure 6:**
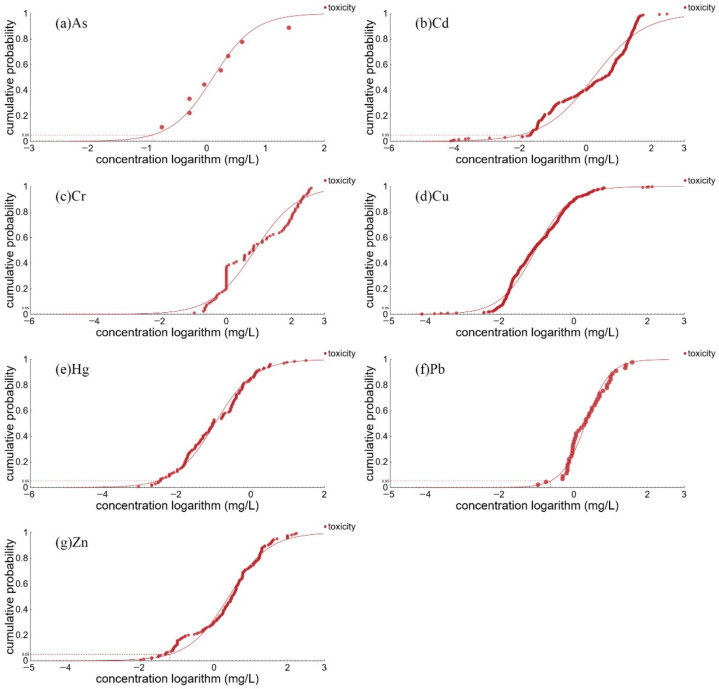
The species sensitivity distribution of heavy metals on coastal Shantou.

The results of the analysis of the potential biohazard effects of heavy metals in Shantou’s sea area by species sensitivity distribution are shown in Figure 6 and Table S4. Among the seven heavy metals, only Cu and Zn pose a defined risk to the ecological environment, and the risk in the spring is higher than in autumn. The other heavy metals do not threaten the local marine life. Wastewater from the toy and electronic manufacturing industries in Shantou contains a variety of heavy metals, including Cu and Zn. Meanwhile, there are several mines in the upper reaches of the Rong River, and metal elements in mine discharges may enter the river through surface runoff, which sinks into the South China Sea through the Rong River [15]. The co-occurrence of Cu and Zn also points to another source of metal pollution, which is marine transportation. Hulls made of alloys often contain Cu and Zn [48], while ballast water or sewage from cargo ships can lead to higher levels of heavy metals [49]. Coupled with the numerous docks in Shantou Bay, aquaculture activities have also become an important source of heavy metals in seawater [19]. The risk of heavy metals in Shantou’s sea area has obvious spatial distribution characteristics, and the potential hazard ratio in the sea area near the coast is significantly higher than that in the sea area further from the coast, which may be caused by the fact that there are more rivers in the estuary, such as Rongjiang River, Lianjiang River and Waisha River.

**Figure 7:**
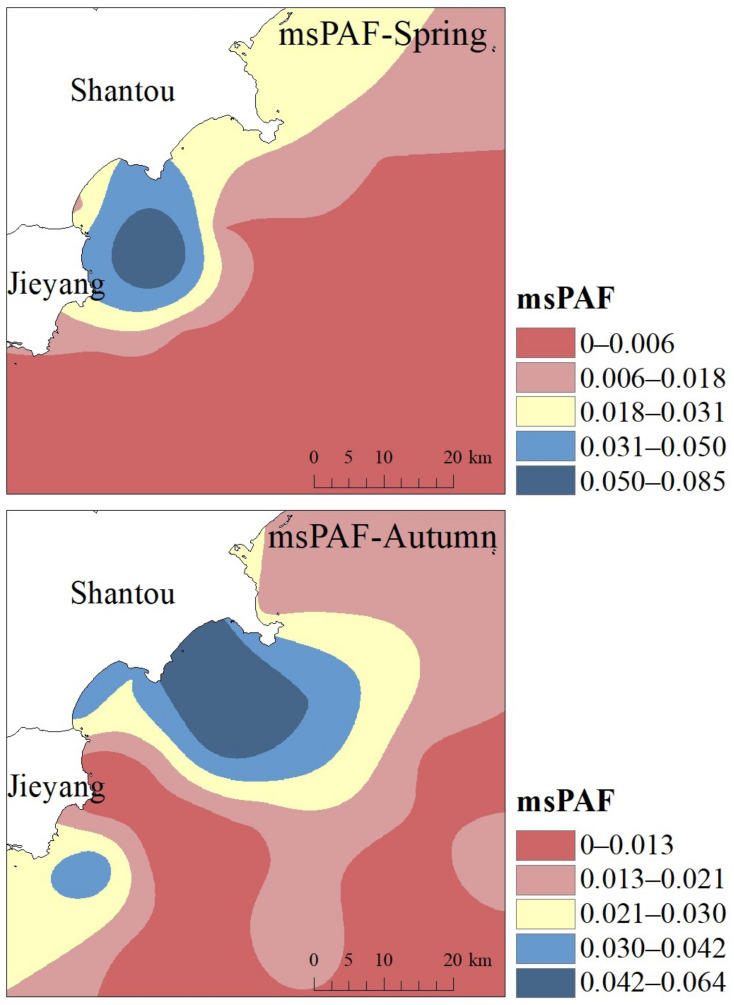
Compound hazard ratio (msPAF) of heavy metals in the seawater of Shantou.

The interpolation diagram of the compound hazard ratio of Shantou’s sea area is shown in Figure 7. The msPAF of Shantou’s sea area has obvious spatial distribution characteristics. The high-value areas all appear in the sea area not far from the coastline and then gradually decrease to the distance of the ocean. In the autumn, the high values of the interpolation plot are close to the coastline. As mentioned above, the industries in the east of Guangdong Province are mainly light industries, coupled with the construction of reclamation projects resulting in the reduction of bay wetlands, more estuaries and other multiple reasons, the distribution of msPAF in Shantou’s sea area has such characteristics. Overall, the compound hazard ratio in Shantou’s sea area is not high, which is in an acceptable range, but protection from heavy metal pollution cannot be ignored. The most direct and powerful way to reduce ecological risk is to control the wastewater discharge of polluting enterprises and carry out effective wastewater treatment of all enterprises.

### 3.5. Priority Control and Recommendations

Heavy metal pollution poses a growing threat to the bay’s aquatic environment. Heavy metals present in the ecosystem are not biodegradable, and their removal from seawater and sediments is extremely difficult and sometimes impossible [1]. Therefore, it is crucial to understand the distribution of different heavy metals in the region and propose corresponding targeted measures to protect the marine ecosystem. The degree of heavy metal pollution in Shantou’s sea area is clean. Cu, Pb, and Hg exceeded the standard in seawater at a few points. Although Cu, as a trace element, is essential to organisms, when it exceeds a certain threshold, it will cause harm to organisms [50]. With accelerated urbanization, the popularization of household cars and the development of industrial enterprises, a lot of fossil fuels are consumed, and Pb is likely to come from these fossil fuels [51]. Hg is notorious for causing tragic poisonings in Japan. Although the current heavy metal pollution of Shantou’s sea area is not serious, it should be prevented in advance.

The heavy metal pollution in Shantou’s sea area mostly comes from anthropogenic factors, such as domestic sewage, industrial wastewater, agricultural surface runoff and shipping pollution. The areas with high pollution value all appear near the coastline. Therefore, the detection scope of marine ecology can be appropriately narrowed, focusing on the nearby coast, which saves a good deal of labor-related and material resources, improves detection efficiency, and obtains more accurate data. Shantou is one of the earliest special economic zones in China. Although the economic development of Shantou is somewhat backward, attention should be paid to protecting and managing the marine environment during development initiatives to ensure high-quality sustainable development.

## 4. Conclusions

The cumulative effects of different heavy metals in different media are very different, so a single evaluation method cannot accurately evaluate the pollution level of heavy metals. By assessing the ecological distribution characteristics of heavy metals and the transport relationship of heavy metals between different media, and combining with species sensitivity distribution assessment, we effectively assessed the concentration levels, spatial and temporal distribution patterns and ecological risks of seven heavy metals. The results showed that the pollution levels of heavy metals in Shantou’s sea area is low, Cu, Pb and Hg exceeded the national first-class water quality standard in a few seawater sites, and only Cu and Zn caused ecological risks. This is related to the level of urbanization, industrial structure and unique geographical location of Shantou city. Although the heavy metal pollution in Shantou’s sea area is not serious, the potential ecological risk of the seven metals should not be ignored, as the *C_f_*, *WQI*, PAF values, and other indicators will all provide early warning. Among the areas investigated, more attention should be paid to the pollution in the areas close to the coastline, especially in bays and estuaries, and effective pollution monitoring and control measures should be formulated. In addition, the development of multiple industries, including aquaculture and shipping, should be coordinated, prevention and control mechanisms should be enforced, and sewage discharge should be strictly controlled to protect the marine ecosystem.

## Figures and Tables

**Table 1:** Results of standard index evaluation.

Carrier	Season	Statistical Parameter	Cu	Pb	Zn	Cd	As	Cr	Hg
Seawater	spring	Max	1.14	1.66	0.49	0.07	0.03	0.05	1.46
		Min	0.02	0.02	0.08	0.01	0.01	<0.01	0.04
		CV	5.00%	5.00%	0	0	0	0	5.00%
	autumn	Max	0.72	1.66	0.59	0.01	0.06	0.01	1.24
		Min	0.01	0.3	0.18	0.01	0.05	<0.01	0.04
		CV	0	35.00%	0	0	0	0	15.00%
Marine organism	spring	Max	0.21	0.03	0.13	0.48	0.34	-	0.18
		Min	0.01	0.01	0.04	0.01	0.01	-	0.01
		CV	0	0	0	0	0	-	0
	autumn	Max	0.18	0.01	0.14	0.62	0.2	-	0.15
		Min	0.03	0.01	0.02	0.01	0.04	-	0.01
		CV	0	0	0	0	0	-	0
Sediments	spring	Max	0.6	0.81	0.7	0.18	0.93	0.6	0.48
		Min	0.01	0.2	0.2	0.04	0.03	0.2	0.14
		CV	0	0	0	0	0	0	0

The seawater index is judged according to the People’s Republic of China Seawater Quality Standards (GB3097-1997); the sediment index is judged according to the Specifications for Oceanographic Survey (GB/T 12763-2007); the marine organism index is judged according to Countrywide Comprehensive Investigations of the Coastal Zone and Tidal Land Resources and Technical Regulations for the Second National Baseline Survey of Marine Pollution.

**Table 2:** Flowability of heavy metals in coastal Shantou.

Season	Spring							Autumn						
Site	Cu	Pb	Zn	Cd	Hg	As	Cr	Cu	Pb	Zn	Cd	Hg	As	Cr
S1	3.00	-	-	-	-	-	-	1.88	16.26	4.57	-	0.44	3.55	-
S3	21.33	463.33	9.93	-	12.89	15.85	43.13	5.33	34.18	8.18	-	2.23	8.65	57.50
S4	19.00	-	11.40	-	16.91	-	-	41.80	36.15	14.77	-	5.47	9.42	-
S5	15.63	-	10.99	-	4.15	-	-	41.67	55.61	12.15	-	1.38	9.18	-
S7	2.53	22.53	12.62	5.00	3.21	24.00	76.40	36.00	32.24	11.20	-	2.26	12.00	-
S8	-	-	20.21	-	1.80	20.80	-	40.67	32.40	13.17	-	1.74	9.45	-
S11	68.50	198.24	-	-	-	-	-	7.21	25.34	8.10	-	1.51	10.36	-
S13	-	143.08	-	-	0.67	10.46	-	15.75	36.47	8.52	-	1.63	5.23	-
S14	-	81.82	-	-	-	-	-	-	19.57	5.37	-	0.38	7.15	-
S16	-	-	-	-	-	8.18	-	-	54.59	6.04	-	0.37	4.46	-
S18	-	272.22	-	-	-	-	5.16	-	48.04	4.18	-	0.23	5.55	-
S20	-	-	-	-	-	-	-	6.40	11.86	6.61	-	-	3.71	-

## Data Availability

Not applicable.

## Supplementary Materials

The following supporting information can be downloaded at: https://www.mdpi.com/article/10.3390/ijerph192316022/s1.
