# Supplementary material for: Pollution Assessment and SSD-Based Ecological Assessment of Heavy Metals in Multimedia in the Coast of Southeast China
Source: Int J Environ Res Public Health. 2022 Nov 30;19(23):16022. doi: 10.3390/ijerph192316022 (PMC9736362; doi:10.3390/ijerph192316022)
Supplement: Supplementary file 1 [file ijerph-19-16022-s001.zip › supplementary material.pdf]

Table S1 Geochemical background values in sedimentary rocks (mg/kg)

elements	Cu	Zn	Pb	Cd	Cr	As	Hg
C_n^i	7.4	54.5	15.6	0.18	39.3	9.7	0.02

Table S2 The classification of E_r^i and PERI

$\mathbf{E_r^i}$	**Ecological risk posed by individual metal**
<40	Low risk
40 - 80	Moderate risk
80 - 160	Considerable risk
160 - 320	High risk
> 320	Very high risk
**PERI**	**Overall ecological risk posed by multiple metals**
< 150	Low risk
150 - 300	Moderate risk
300 - 600	Considerable risk
> 600	Very high risk

Table S3 The People 's Republic of China national standard on heavy metal concentration (mg/L or mg/kg)

Carrier	Water grades		Cu	Pb	Zn	Cd	As	Cr	Hg
Seawater	I		0.005	0.001	0.02	0.001	0.02	0.05	0.00005
	II		0.010	0.005	0.050	0.005	0.030	0.10	0.0002
	III		0.050	0.010	0.10	0.010	0.050	0.20	0.0002
Marine organism	Shellfish	I	10	0.2	20	0.2	1.0	0.5	0.05
		II	25	2	50	2	5	2	0.1
		III	50	6	100	5	8	6	0.3
	Fish	I	20	2	40	0.6	5	1.5	0.3
	Crustaceans	I	100	2	150	2	8	1.2	0.2
	Mollusc	I	100	10	250	5.5	8	1.5	0.3
Sediments	I		35	60	150	0.5	20	80	0.2
	II		100	130	350	1.5	65	150	0.5
	III		200	250	600	5	93	270	1

Seawater evaluation implements the first class of the People’s Republic of China Seawater Quality Standards (GB3097-1997); sediment evaluation standards implement the first class of the People’s Republic of China Marine Sediment Quality Standards (18668-2002); the evaluation standard of fish and crustacean samples adopts the biological quality standard stipulated in the Concise Regulation for Comprehensive Investigation of National Coastal and Tidal Flat Resources, and the shellfish samples adopt the first class standard in the National Standard of the People 's Republic of China Marine Biological Quality (GB 18421-2001).

Table S4 I_{geo} and E_r^i statistical results of sediments

		Cu	Pb	Zn	Cd	Hg	As	Cr
I_{geo}	Max	0.91	1.06	0.36	-1.58	2.63	-0.28	-0.61
	Min	-1.99	-0.95	-1.46	-2.43	-2.32	-1.90	-2.37
	Average	-0.38	0.13	-0.49	-2.22	-0.02	-0.96	-1.34
E_r^i	Max	14.12	15.64	1.92	15.00	372.00	12.37	1.96
	Min	1.89	3.88	0.54	8.33	12.00	4.02	0.58
	Average	7.31	8.95	1.18	10.00	101.50	8.32	1.30

Table S5 Detection results of heavy metals in marine organisms

season	station	species	Hg	Cu	Pb	Zn	Cd	As
Spring	SY1	Oratosquilla oratoria	0.02	21.3	<0.04	19.2	0.953	1.3
	SY2	Polinices didyma	0.02	<2.0	<0.04	16.2	0.008	0.8
	SY2	Neptunea	<0.01	<2.0	<0.04	9	0.066	0.8
	SY3	Parapenaeopsis hardwickii	0.01	6.9	<0.04	12.5	0.011	0.7
	SY4	Portunus gladiator	<0.01	7.4	<0.04	18.5	0.089	2.8
	SY5	Evynnis cardinalis	<0.01	<2.0	<0.04	3.2	0.012	0.6
	SY6	Black-finned cardinalfish	0.03	<2.0	<0.04	2.2	0.011	0.5
	SY6	Upeneus bensasi	0.01	<2.0	0.07	2	0.016	0.6
	SY7	Saurida undosquamis	0.03	<2.0	<0.04	2.9	0.018	<0.2
	SY8	Trachiocephalus myops	0.05	<2.0	<0.04	4.7	0.016	0.3
	SY9	Rosed razorfish	0.04	<2.0	<0.04	2.8	0.012	0.4
	SY12	Uroteuthis duvauceli	<0.01	<2.0	<0.04	8.7	0.32	0.8
	average		0.03	11.9	0.07	8.5	0.128	0.9
	detectable rate(%)		66.67	25	8.33	100	100	91.67
Autumn	SY1	Lepturacanthus savala	0.04	<2.0	<0.04	2.2	0.005	0.5
	SY2	Little Spinefoot	0.01	<2.0	<0.04	3	<0.005	0.5
	SY3	Solenocera melantho	0.02	2.9	<0.04	8.5	<0.005	0.7
	SY4	Portunus sanguinolentus	<0.01	6.4	<0.04	10.3	0.048	0.8
	SY5	Japanese threadfin bream	0.02	<2.0	<0.04	2.3	0.005	0.5
	SY6	Leiognathus berbis	0.01	<2.0	<0.04	5.6	0.007	0.6
	SY7	Setipinna tenuifilis	<0.01	<2.0	<0.04	2.9	0.01	0.4
	SY7	Uroteuthischinensis	0.02	<2.0	<0.04	7.2	0.042	0.6
	SY8	Uroconger	0.03	<2.0	<0.04	3.1	0.013	0.6

	SY9	Sepioteuthis lessoniana	0.02	3.4	<0.04	12.3	0.009	0.7
	SY9	Oratosquilla oratoria	0.02	17.8	<0.04	12.4	0.369	1.4
	SY10	Johnius belangerii	0.02	<2.0	<0.04	1.6	<0.005	0.3
	SY10	Trypauchen	0.02	<2.0	<0.04	4.1	<0.005	1
	SY11	Muraenesox cinereus	0.04	<2.0	<0.04	3.9	<0.005	0.6
	SY12	Pennahia pawak	0.04	<2.0	<0.04	0.7	<0.005	0.2
	average		0.02	7.6	-	5.3	0.056	0.6
	detectable rate(%)		86.67	26.67	0	100	60	100

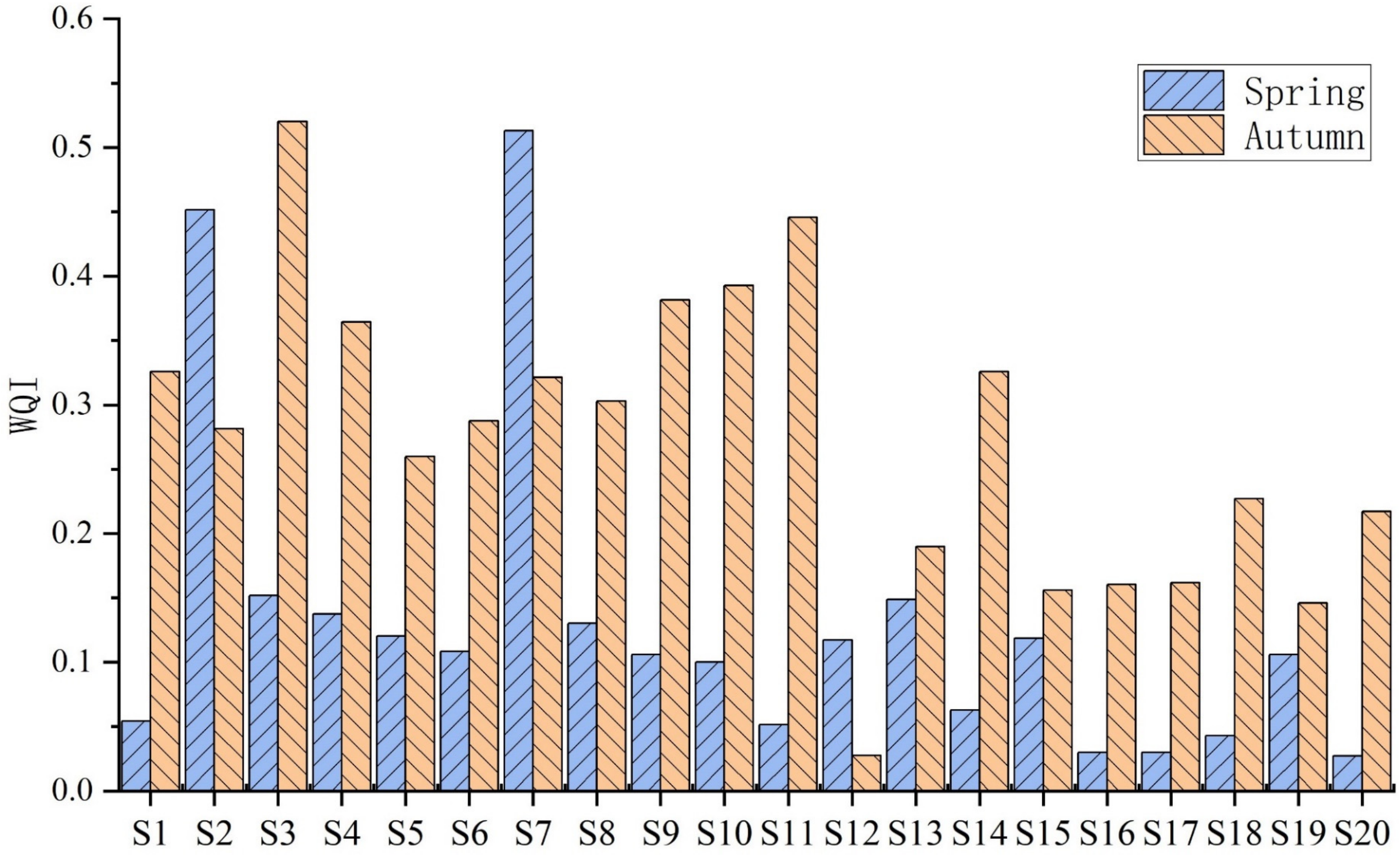

Figure S1. WQI of heavy metals in surface seawater of Shantou.

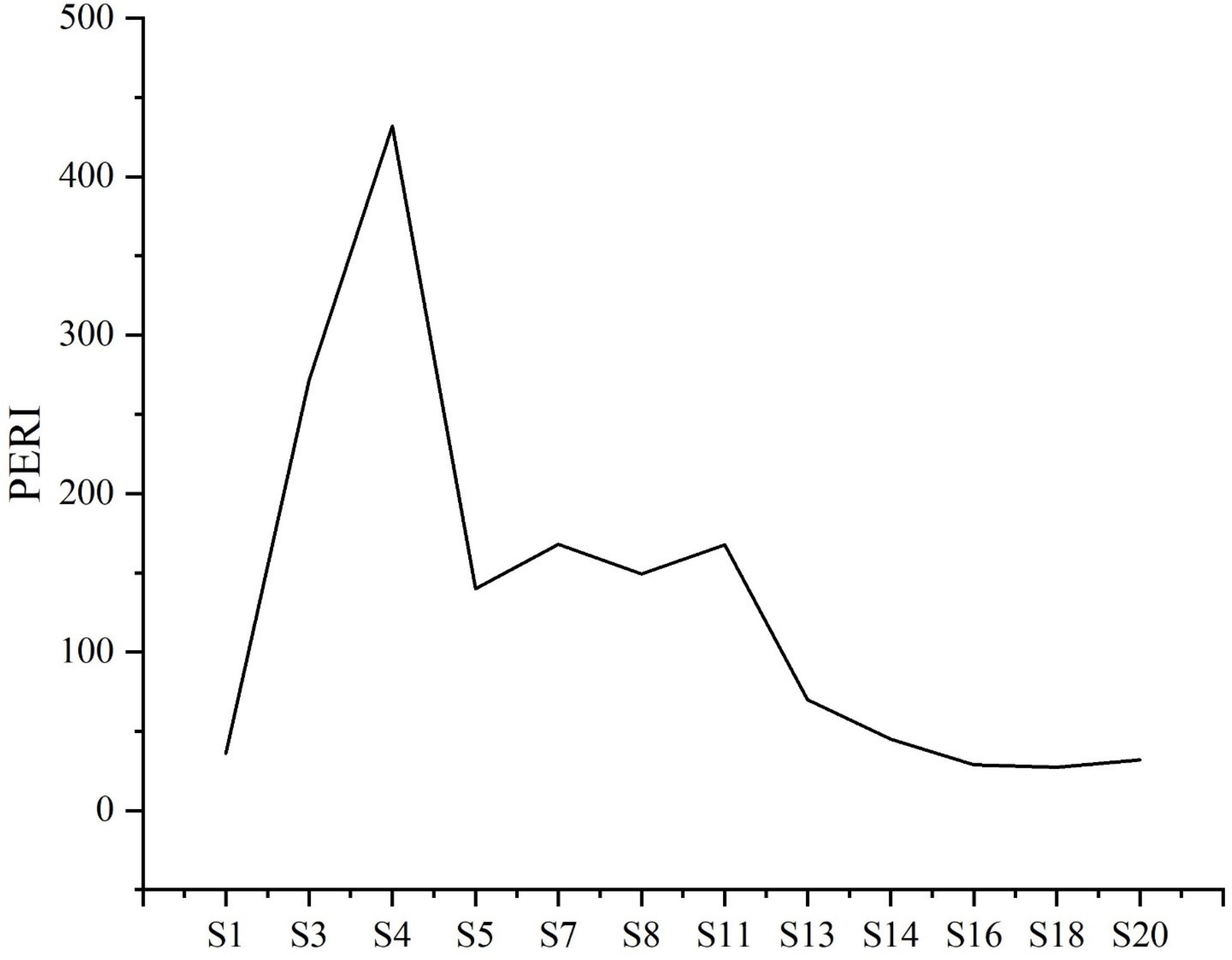

Figure S2. The results of PERI in coastal of Shantou.
